# Functional Connectivity of Nucleus Accumbens and Medial Prefrontal Cortex With Other Brain Regions During Early-Abstinence Is Associated With Alcohol Dependence and Relapse: A Resting-Functional Magnetic Resonance Imaging Study

**DOI:** 10.3389/fpsyt.2021.609458

**Published:** 2021-01-28

**Authors:** Xia Yang, Ya-jing Meng, Yu-jie Tao, Ren-hao Deng, Hui-yao Wang, Xiao-jing Li, Wei Wei, Yu Hua, Qiang Wang, Wei Deng, Lian-sheng Zhao, Xiao-hong Ma, Ming-li Li, Jia-jun Xu, Jing Li, Yan-song Liu, Zhen Tang, Xiang-dong Du, Jeremy W. Coid, Andrew J. Greenshaw, Tao Li, Wan-jun Guo

**Affiliations:** ^1^Mental Health Center and Psychiatric Laboratory, The State Key Laboratory of Biotherapy, West China Hospital of Sichuan University, Chengdu, China; ^2^Department of Clinical Psychology, Suzhou Psychiatric Hospital, The Affiliated Guangji Hospital of Soochow University, Suzhou, China; ^3^Department of Psychiatry, University of Alberta, Edmonton, AB, Canada; ^4^Center for Educational and Health Psychology, Sichuan University, Chengdu, China

**Keywords:** alcohol dependence, relapse, relapse severity, predictor, rest-functional magnetic resonance imaging, functional connectivity

## Abstract

**Background:** Alcohol dependence (AD) is a chronic recurrent brain disease that causes a heavy disease burden worldwide, partly due to high relapse rates after detoxification. Verified biomarkers are not available for AD and its relapse, although the nucleus accumbens (NAc) and medial prefrontal cortex (mPFC) may play important roles in the mechanism of addiction. This study investigated AD- and relapse-associated functional connectivity (FC) of the NAc and mPFC with other brain regions during early abstinence.

**Methods:** Sixty-eight hospitalized early-abstinence AD male patients and 68 age- and education-matched healthy controls (HCs) underwent resting-functional magnetic resonance imaging (r-fMRI). Using the NAc and mPFC as seeds, we calculated changes in FC between the seeds and other brain regions. Over a follow-up period of 6 months, patients were measured with the Alcohol Use Disorder Identification Test (AUDIT) scale to identify relapse outcomes (AUDIT ≥ 8).

**Results:** Thirty-five (52.24%) of the AD patients relapsed during the follow-up period. AD displayed lower FC of the left fusiform, bilateral temporal superior and right postcentral regions with the NAc and lower FC of the right temporal inferior, bilateral temporal superior, and left cingulate anterior regions with the mPFC compared to controls. Among these FC changes, lower FC between the NAc and left fusiform, lower FC between the mPFC and left cingulate anterior cortex, and smoking status were independently associated with AD. Subjects in relapse exhibited lower FC of the right cingulate anterior cortex with NAc and of the left calcarine sulcus with mPFC compared to non-relapsed subjects; both of these reductions in FC independently predicted relapse. Additionally, FC between the mPFC and right frontal superior gyrus, as well as years of education, independently predicted relapse severity.

**Conclusion:** This study found that values of FC between selected seeds (i.e., the NAc and the mPFC) and some other reward- and/or impulse-control-related brain regions were associated with AD and relapse; these FC values could be potential biomarkers of AD or for prediction of relapse. These findings may help to guide further research on the neurobiology of AD and other addictive disorders.

## Introduction

Alcohol use disorder (AUD) is a major burden on public health. It not only contributes to physical diseases and mental disorders but also increases the incidence of traffic accidents and impulsive violence (1). Approximately 240 million adult people worldwide are estimated to suffer from AUD (2). Globally, alcohol may cause 5.3% of all deaths and 5.1% of disability-adjusted life years (DALYs) lost (3), and is the 3rd highest risk factor of disease and 7th highest risk factor when considering both deaths and DALYs together (4). According to the criteria of the DSM-IV, AUD may be categorized as either alcohol dependence (AD) or alcohol abuse. A systematic review showed that for males, the overall lifetime and current prevalence of AD in mainland China were 4.7 and 4.4%, respectively; for females, the corresponding data were below 0.1% (5). One of the main reasons for its large disease burden worldwide is that AD is a chronic encephalopathy with a high relapse rate (6). Prospective studies indicated that the relapse of AD is high (i.e., higher than 60% in most studies) (7, 8).

Consequently, there is an urgent need to explore the predictive factors for development and progression of AD, including pathogenesis and relapse. However, most studies on the diagnosis and prognosis of AD have focused on clinical features, such as drinking history, clinical symptoms, and compliance with treatment (9–11). These studies documented inconsistent evidence and did not adequately clarify the neurobiological etiology of AD and its relapse.

The rapid development of the non-invasive technique of magnetic resonance imaging (MRI), with associated advantages of safety, and high resolution, has provided an excellent window for studying brain mechanisms of mental disorders *in vivo*, including addiction (12, 13). When neuroimaging studies began, most morphometric studies observed that the structure of gray and white matter had abnormal changes in subject with AD (14). Meta-analysis results indicated, among all gray matter damage, a significant reduction in corticostriatal-limbic loops, such as the striatum, dorsal lateral prefrontal cortex, and anterior cingulate cortex. Gray matter reduction in nucleus accumbens (NAc) region was negatively associated with the duration of AD, while shrinkage of mPFC regions was related to lifetime drinking consumption (15). Some studies have gradually focused on functional MRI (fMRI) studies. For example, Cservenka et al. (16) reported that youth with a family history of alcoholism differed from healthy controls (HCs) with respect to functional connectivity (FC) between NAc and medial prefrontal cortex (mPFC) (16). Wrase et al. observed lower activation of the NAc region in the execution of a monetary gain task and increased activation in alcohol-related cues among AD patients but not HCs (17). These cross-sectional neuroimaging studies documented evidence that both structural and functional changes in some brain regions, especially in regions of frontostriatal circuits (including the NAc and mPFC), are associated with AD. Some reviews of the mechanisms of addiction in neuroimaging, including AD, suggested that the NAc and mPFC may play a key role in adjusting the balance between goal-directed, craving, habit and, compulsion on the macroscale level (18). Meanwhile, on the mesoscale or microscale levels of tracing neurons and synapses, alterations of corticostriatal-limbic systems, including mesolimbic glutamate, dopamine, and gamma-amino-butyric acid (GABA) pathways, are associated with addictive behavior (19). Disrupted function of reward circuity is proposed to be a key mechanism of AD and other forms of addiction (20). It is widely accepted that the NAc plays a crucial role in the reward circuit (21). With the development of addiction, changes in the reward pathway are shifted from reward-driven behavior at the early stage to loss of reward at the later stage (22). Meanwhile, other models of addiction suggested that substance use is initially goal-directed behavior, aiming at improving sleep, regulating emotion or prompting relationships with others, and finally develops into habitual or compulsive behavior (23). The changes from goal-directed to compulsive behavior may result from the structure or function of the brain after long-lasting drug use. The NAc and mPFC have been regarded as crucial roles in the reward-learning-execution pathway (24).

Relatively few studies have investigated brain functional connectivity patterns to predict future relapse. Beck et al. found, for the alcohol-cue stimuli task in relapsers compared with abstainers, lower FC between the midbrain and the left mPFC (25). Cope et al. used a survey to show that higher activation in the NAC could predispose individuals to earlier substance use during 3 years follow-up (26). Camchong et al. suggested that, compared to abstainers, diminished resting-state synchrony of relapsers in execution of the Go-No-go task between the executive control networks, reward circuit and visual network may predict relapse after a 6-month follow-up period (27). These longitudinal studies mainly focused on task-status fMRI and determined the importance of the NAc and mPFC to relapse prognoses, while some researchers indicated that alcohol craving not only appears in response to alcohol-cue stimuli but also occurs in a resting state; thus, further study of the changes in rest-fMRI (r-fMRI) is needed (28, 29).

Considering the importance of the NAc and mPFC in the reward circuit and in the cognitive execution function of addiction, based on prior evidence implicating the NAc and mPFC as regions of interest (ROIs) or seeds, this study aimed to investigate the AD- and relapse-associated FC changes with other brain regions during early abstinence using a 6-month follow-up longitudinal design.

## Materials and Methods

This longitudinal cohort study was conducted in the mental health center of a general hospital from September 2015 to September 2019 and approved by the Ethics Committee of West China Hospital of Sichuan University in 2016 (NO.22). Informed written consent was obtained from every participant in this study.

### Participants

One hundred thirty-six male participants (68 alcohol-dependent patients and 68 HCs) enrolled in this study. All participants were all right-handed and of Han nationality. All recruited patients experienced hospitalized detoxification and were in the early stage of abstinence. Each patient met DSM-IV criteria of AD according to the Structured Clinical Interview for DSM-IV Axis I Disorders-Patient Edition (SCID-I/P) administered by psychiatric clinicians (30). During screening, participants provided information about their past, current, and recent drinking, including consumption quantity and frequency. Exclusion criteria for AD cases in this study were a history of neurological illness (or trauma) and a history of past or present substance use disorders except alcohol or tobacco dependence, intelligence quotient <70, age of <18 years and or more than 55 years. Matched health control subjects in age and education were also recruited from the general community. HCs were assessed and selected with the diagnostic interview of DSM-IV Axis I Disorders-Nonpatient Edition (SCID-NP). The exclusion criteria for controls were psychotic disorders, emotional disorders, substance-use disorders except tobacco dependence, or having a first-degree relative with psychotic disorders.

### Baseline Measurements

A series of self-conducted questionnaires were used to collect demographic information (including age, education years, and smoking status) and clinical characteristics [including data related to alcohol use (including the age of the participant's first drink and years of alcohol use)].

Alcohol use in recent years was measured using a Chinese version of the Alcohol Use Disorder Identification Test (AUDIT). The AUDIT is a short screening tool consisting of 10 questions (total score ranged from 0 to 40) to estimate severity of alcohol consumption in the preceding year (31, 32). Different language versions (including a Chinese version) have been validated worldwide (33).

MRI scans were acquired during early abstinence (5–12 days after stopping drinking) using a 3.0 T (Achieva; Philips, Amsterdam, the Netherlands) scanner equipped with an *Invivo* HD eight-channel high-resolution head coil. Foam padding and earplugs were used to minimize head movement and scanner noise. During examination, participants were instructed to rest quietly with their eyes closed and to keep their minds blank without falling asleep (confirmed by participants immediately after finishing MRI scans).

Approximately 8 min of resting-state echo-planar imaging (EPI) scans were acquired for each participant with 38 axial slices and 240 volumes (TR = 2.0 s, TE = 3.0 ms; flip angle = 90°; matrix 256 × 256; FOV 24 × 24 cm^2^; and voxel size = 3.75 × 3.75 × 4 mm^3^). High-resolution T1-weighted anatomical images with 188 contiguous axial 1 mm thick slices were also acquired (TR = 8.1 ms, TE = 3.7 ms; flip angle = 7°; matrix 256 × 256; and FOV 25.6 × 25.6 cm^2^).

### Outcomes

AD patients were regularly followed up at 1, 3, and 6 months after the baseline survey. During the follow-up interview, all participants were asked “Have you had alcohol again since the latest discharge from the detoxification hospital?” Subjects who replied “yes” were further interviewed for their time (months) of the first drink after discharge and alcohol consumption of the most severe month after discharge using a revised Chinese 1-month version of AUDIT (34). To assess the relapse severity in the month with the heaviest drinking after the baseline or after the last follow-up interview, the timeframe of the scale was revised from 1 year to 1 month. According to previous studies on the optimal cutoff of the AUDIT score to screen for alcohol use disorders (31, 33), previously detoxified patients were categorized into the relapse group if the total AUDIT score was ≥8 in any follow-up interviews, and other patients were included in the non-relapse group.

### Imaging Data Acquisition

#### Functional Connectivity Analyses

Baseline imaging data were preprocessed by the Data Processing Assistant for Resting-State fMRI (DPARSF) (35) software toolbox in MATLAB (MathWorks, Natick, MA, USA). After removing the first ten time points, the data were slice-time corrected, realigned and normalized to the Montreal Neurological Institute (MNI) template and resampled to 3 × 3 × 3 mm^3^. Nuisance covariates containing Friston-24 motion parameters, white matter and cerebrospinal fluid (CSF) were regressed out (36). The residual time series underwent bandpass filtering within a frequency range of 0.01–0.10 Hz (37), which reflected mainly neuronal oscillations and could eliminate high-frequency physiological noise. Data were smoothed with a Gaussian kernel (full-width half-maximum: 6 6 6 mm). Head motion parameters of all images were <1.5 mm, and the rotation was <1.5°. No significant differences between groups were calculated for framewise displacement, in line with Power et al. (38). FC is one type of processed r-fMRI data that can be used to examine the correlation between a seed voxel and other voxels in the whole brain. The NAc (Talairach coordinates x = 0, y = 10, z = −10, radius = 10 mm) (39) and mPFC (Talairach coordinates x = 0, y = 42, z = 18, r = 20 mm) (40) were defined as seeds to explore FC changes in relation to the rest of the whole brain in a voxel-wise manner.

### Statistical Analyses

#### Imaging Data Comparison

Two sample *t*-tests contained in DPARSF were used to estimate the differences in baseline FC values between alcohol-dependent patients and HCs, and between the relapse and the non-relapse group, respectively. The confounding effects of age, education years, and smoking status were adjusted as covariates. Additionally, to identify the association between FC and the symptom severity of relapse (AUDIT scores) in the relapse group, a series of partial correlation analyses was conducted in DPARSF by adjusting for age, education years, and smoking status. Statistical inferences were made with *p* < 0.001 for multiple comparisons at the voxel level, adjusted using cluster-level threshold familywise error (FWE) based on Gaussian Random Field; *p* < 0.05 indicated statistical significance for bidirectional (i.e., positive and negative) explorations.

#### Logistic Regression

Each participant's baseline FC values that were significantly different between patients with AD and controls or between relapse- and non-relapse category participants, were extracted for further logistic regression to determine FC indictors independently associated with AD or independently predictive of relapse. Binary logistic regression (forward: conditional) was performed using demographic data including age and education years, clinical characteristics (including smoking status and, for relapse category participants, baseline AUDIT scores), as well as extracted FC values as independent variables. To represent the classifying or predictive power of the regression models and each FC independently associated with AD or relapse, the area under the receiver operating characteristic (ROC) curve (AUC) was employed in the next steps.

#### Linear Regression

Each patient's baseline FC values, which were significantly correlated with the severity of relapse in partial correlation analysis in the relapse group, were extracted for further linear regression to determine FC indictors independently associated with the severity of relapse. Multivariate linear regression (stepwise) was conducted using demographic data (age and education years), clinical characteristics (smoking status and baseline AUDIT scores) and the correlated FC values as independent variables.

The rates (95% confidence intervals, 95% CI) and means (±standard deviation, SD) of demographic variables and outcomes were used to derive descriptive statistics. Student's *t*-tests for quantitative data and χ^2^ tests for categorical data were used to calculate differences between groups. Regression analyses outlined above were conducted in SPSS 25.0 (IBM Corp., Armonk, NY, USA) using a two-tailed α level criterion of 0.05.

## Results

### Sample Characteristics

The sample characteristics are illustrated by the data displayed in Table 1. Age (AD group: 39.97 ± 9.00 years; HC group: 38.03 ± 9.53 years) and education years (AD group: 12.81 ± 3.42; HC group: 13.91 ± 3.54) were equivalent between AD and HC groups. The rate of smoking among AD cases (91.04%) was higher than that of HC (38.24%) (χ^2^ = 41.73; *P* < 0.001). Possible confounding effects of age, education years, and smoking status were adjusted as covariates. Mean first drinking age and drinking years of AD were 19.65 ± 5.40 and 20.79 ± 8.71 years. Among AD patients 15 individuals (22.06%) had a positive family history of drinking. The mean AUDIT score of patients with AD at the baseline survey was 28.13 ± 8.22. Based on the 6-month follow-up survey, 35 patients (52.24%) relapsed. Among 32 non-relapsers, there were 28 cases with AUDIT scores of 0 and 4 cases with AUDIT score of 1–6. No significant differences were estimated in age, education years, smoking status or the clinical characteristics mentioned above, between the relapse and non-relapse groups. As expected, the most severe AUDIT score at the follow-up survey was higher in the relapse group than observed in the non-relapse group.

**Table 1:** Sample demographics and their clinical characteristics based on baseline- and follow-up surveys.

	**AD^a^ (*n* = 68)**	**HC^b^ (*n* = 68)**		**Relapse^c^ (*n* = 35)**	**Non-relapse^d^ (*n* = 32)**	
	**Mean ± SD^e^**	**Mean ± SD^e^**	***t*^f^/χ^**2**^*^g^***	**Mean ± SD^e^**	**Mean ± SD^e^**	***t*^f^/χ^**2**^*^g^***
**Demographic variables**						
Age (years)	39.97 ± 9.00	38.03 ± 9.53	−1.22	40.97 ± 9.18	38.97 ± 8.95	0.90
Education years	12.81 ± 3.42	13.91 ± 3.54	1.85	12.51 ± 3.41	13.09 ± 3.51	−0.69
Smoking status (%)	62 (91.04%)	26 (38.24%)	41.73^***^	31 (88.57%)	30 (93.75%)	0.55
**Clinical characteristics**						
First drinking age	19.65 ± 5.40			18.71 ± 5.16	20.88 ± 5.45	−1.67
Years of drinking	20.79 ± 8.71			22.36 ± 8.79	18.50 ± 8.38	−1.51
Family history of alcohol drinking (%)	15 (22.06%)			7 (20.00%)	8 (25.00%)	0.24
Consumption in the last month (g)	158.92 ± 70.91			168.40 ± 65.45	144.24 ± 77.67	−0.27
Consumption at the last drinking period (g)	95.11 ± 66.58			22.17 ± 3.62	23.54 ± 3.30	−1.81
AUDIT^h^ score based on baseline survey	28.13 ± 8.22			28.36 ± 8.01	27.96 ± 8.77	−1.85
AUDIT score based on follow-up survey	–			25.26 ± 10.23	0.44 ± 1.34	13.62^***^

a *AD: alcohol dependence*;

b *HC: healthy controls*;

c *Relapse: those who relapsed among alcohol-dependent individuals and had AUDIT scores ≥ 8 at the follow-up survey*;

d *Non-relapse: those who did not relapse among alcohol-dependent individuals and had AUDIT scores <8 at the follow-up survey*.

e *SD: standard deviation*;

f *t: values of student's t-test*;

g *χ^2^: values of Chi-square test*;

h *AUDIT: Alcohol Use Disorder Identification Test*.

*** *P < 0.001*.

### FC Associated With AD

**Figure 1:**
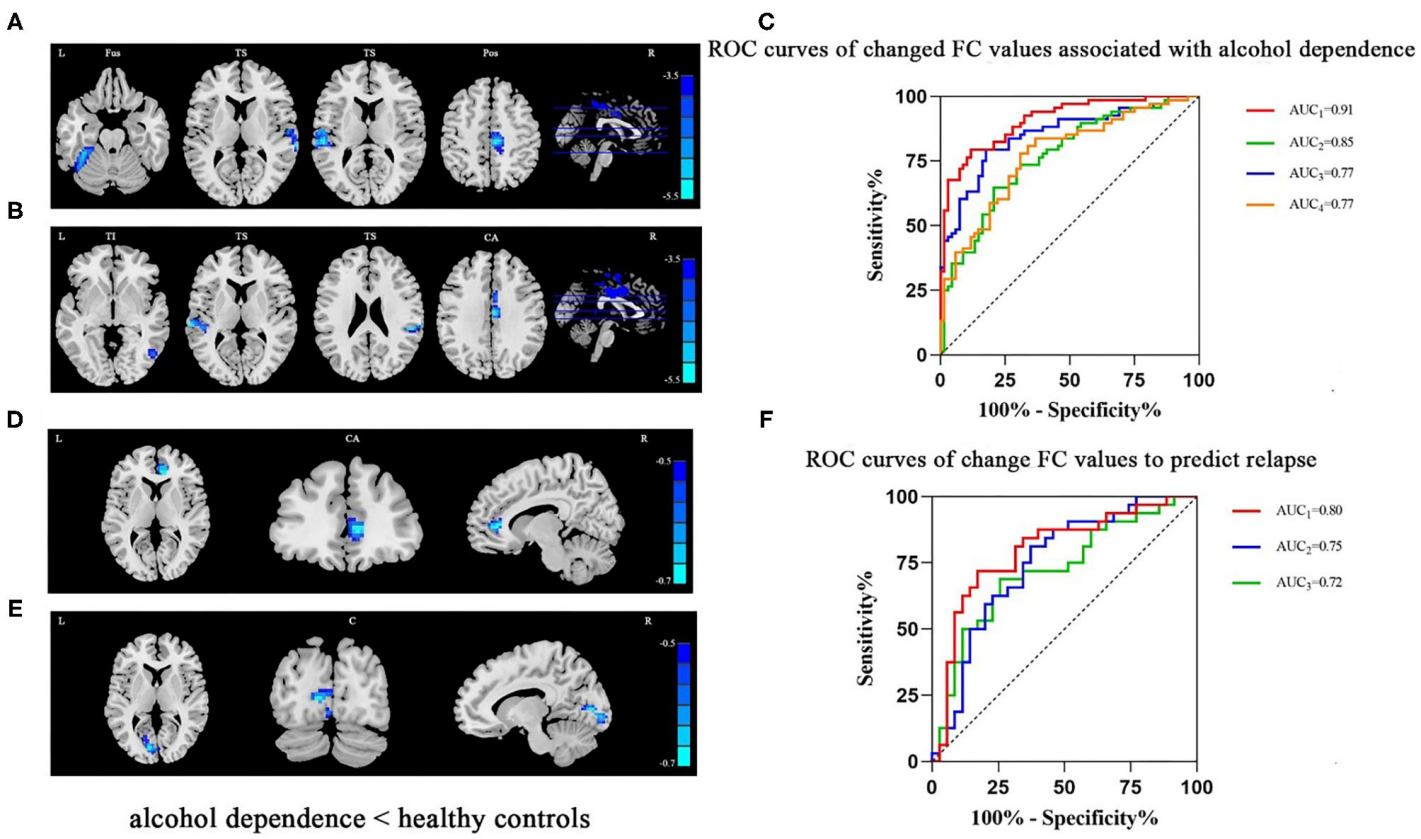
**(A)** In patients with AD, lower FC was detected in the left fusiform, bilateral temporal superior and right postcentral areas (seed: NAc). **(B)** In patients with AD, lower FC was detected in the right temporal inferior, bilateral temporal superior and left cingulum anterior areas (seed: mPFC). **(C)** The ROC curves of logistic regression models using all three indicators (smoking status and two FC indicators, AUC_1_ = 0.91) or two FC indicators (AUC_2_ = 0.85), only FC between the NAc and left fusiform (AUC_3_ = 0.77), or only FC between the mPFC and left cingulum anterior (AUC_3_ = 0.77) to differentiate AD cases and HCs. **(D)** Among patients with AD who had relapsed based on the follow-up survey, lower FC in the right cingulum anterior (seed: NAc) was detected. **(E)** Among patients with AD who had relapsed, lower FC in the left calcarine (seed: mPFC) was detected. **(F)** The ROC curves of logistic regression models using both FCs (AUC_1_ = 0.80), only FC between NAc and right cingulum anterior (AUC_2_ = 0.75), or only FC between mPFC and left calcarine sulcus (AUC_3_ = 0.72) to predict relapse. Clusters were labeled with the Automated Anatomical Labeling (AAL) atlas. The *t* maps of **(A,B)** were drawn with a threshold of *p* < 0.001 at the voxel level and *P*_FWE_ < 0.05 at the cluster level. Though the results of **(D,E)** could not exist relative to the above threshold, *t* maps of these results were shown with a threshold of *p* < 0.016 at the voxel level and *P*_FWE_ < 0.05 at cluster level. The color bar indicates voxel-wise *t* values. lower FC is shown in blue. The brighter the blue, the lower the FC. FC, functional connectivity; NAc, nucleus accumbens; mPFC, medial prefrontal cortex; L, left; R, right; Fus, fusiform; TS, temporal superior gyrus; Pos, postcentral; TI, temporal inferior; CA, cingulum anterior; C, calcarine sulcus; ROC, receiver operating characteristic; AUC, area under curve; AD, alcohol dependence.

Compared to HCs, the FC values of seeds of the NAc with the left fusiform (*t* = −5.27, x = −33, y = −39, z = −24, *P*_FWE_ < 0.001), bilateral temporal superior gyrus (*t* = −4.53, x = 36, y = −9, z = 3, and *t* = −4.76, x = −54, y = −24, z = 15, *P*_FWE_ < 0.001) and right postcentral (*t* = −5.07, x = 9, y = −24, z = 48, *P*_FWE_ < 0.001) (Figure 1A; Supplementary Table 1), and the FCs of seeds of the mPFC with the right temporal inferior (*t* = −4.62, x = 45, y = −45, z = −3, *P*_FWE_ < 0.001), bilateral temporal superior gyrus (*t* = −5.37, x = −66, y = −30, z = 9 and *t* = −5, z = 51, y = −30, z = 24, *P*_FWE_ < 0.001) and left cingulum anterior (*t* = −5.46, x = 3, y = −12, z = 33, *P*_FWE_ < 0.001) of the AD group lower significantly (Figure 1B; Supplementary Table 2). Logistic regression revealed lower FC between the NAc and left fusiform cortex. In addition, lower FC was evident between mPFC and left cingulate anterior cortex, and smoking status - both were independently associated with AD. The AUCs (95% CI) of logistic regression models using all three indicators, two FC indicators, only the FC between the NAc and left fusiform, or only the FC between the mPFC and left cingulate anterior cortex to differentiate AD cases and HCs were 0.91 (0.86, 0.96), 0.85 (0.78, 0.92), 0.77 (0.69, 0.85), and 0.77 (0.70, 0.85), respectively (Table 2; Figure 1C).

**Table 2:** Multivariate logistic regression models using three indicators to AD (*n* = 136).

**Variables**	**β**	**S.E**.	**Wald**	**OR^a^ (95% CI^b^)**
Smoking status	2.90^***^	0.57	25.87	18.24 (5.96, 55.83)
NAc_fusiform_L	−6.31^**^	1.89	11.14	0.002 (0.001, 0.07)
mPFC_cingulum anterior_L	−4.16^**^	1.38	9.13	0.02 (0.001, 0.23)

a *OR: adjusted odds ratio which was based on binary logistic regression (forward: conditional) of alcohol dependence (yes vs. no) as a dependent variable, and age, education years, changes of FC as independent variables*;

b *95% CI: 95% confidence interval; AD, alcohol dependence; NAc, nucleus accumbens; mPFC, medial prefrontal cortex; L, left; R, right*;

** *P < 0.01*;

*** *P < 0.001*.

### FC Values Predicted AD Relapse

Compared with the non-relapse category, the baseline FC values between the NAc and right cingulate anterior cortex seeds (*t* = −3.95, x = 9, y = 39, z = 6, *P*_FWE_ < 0.016) (Figure 1D; Supplementary Table 3) and between the mPFC and left calcarine sulcus seeds (*t* = −3.79, x = −12, y = −81, z = 6, *P*_FWE_ < 0.016) (Figure 1E; Supplementary Table 4) of the relapse group were significantly lower. Logistic regression revealed that both of these FC values independently predicted AD relapse. Based on the AUCs (95% CI) of logistic regression models using both FC values, the FC value between NAc and right cingulate anterior cortex, and the FC value between mPFC and left calcarine sulcus to relapse were 0.80 (95% CI: 0.69, 0.91), 0.75 (0.63, 0.87), and 0.72 (0.60, 0.85), respectively (Table 3; Figure 1F).

**Table 3:** Multivariate logistic regression models to predict relapse (*n* = 67).

**Variables**	**β**	**S.E**.	**Wald**	**OR^a^ (95% CI^b^)**
NAC_cingulum anterior_R	−3.40^*^	1.60	6.26	0.02 (0.001, 0.42)
mPFC_calcarine_L	−3.59^*^	1.53	5.55	0.03 (0.001, 0.55)

a *OR: adjusted odds ratio which was based on the binary logistic regression (forward: conditional) using relapse (yes vs. no) as a dependent variable, and age, education years, AUDIT scores at baseline survey and FC changes as independent variables*;

b *95% CI: 95% confidence interval; NAc, nucleus accumbens; mPFC, medial prefrontal cortex; L, left; R, right*;

* *P < 0.05*.

### Results of FC Values Correlated With Relapse Severity and Its Predictors

**Figure 2:**
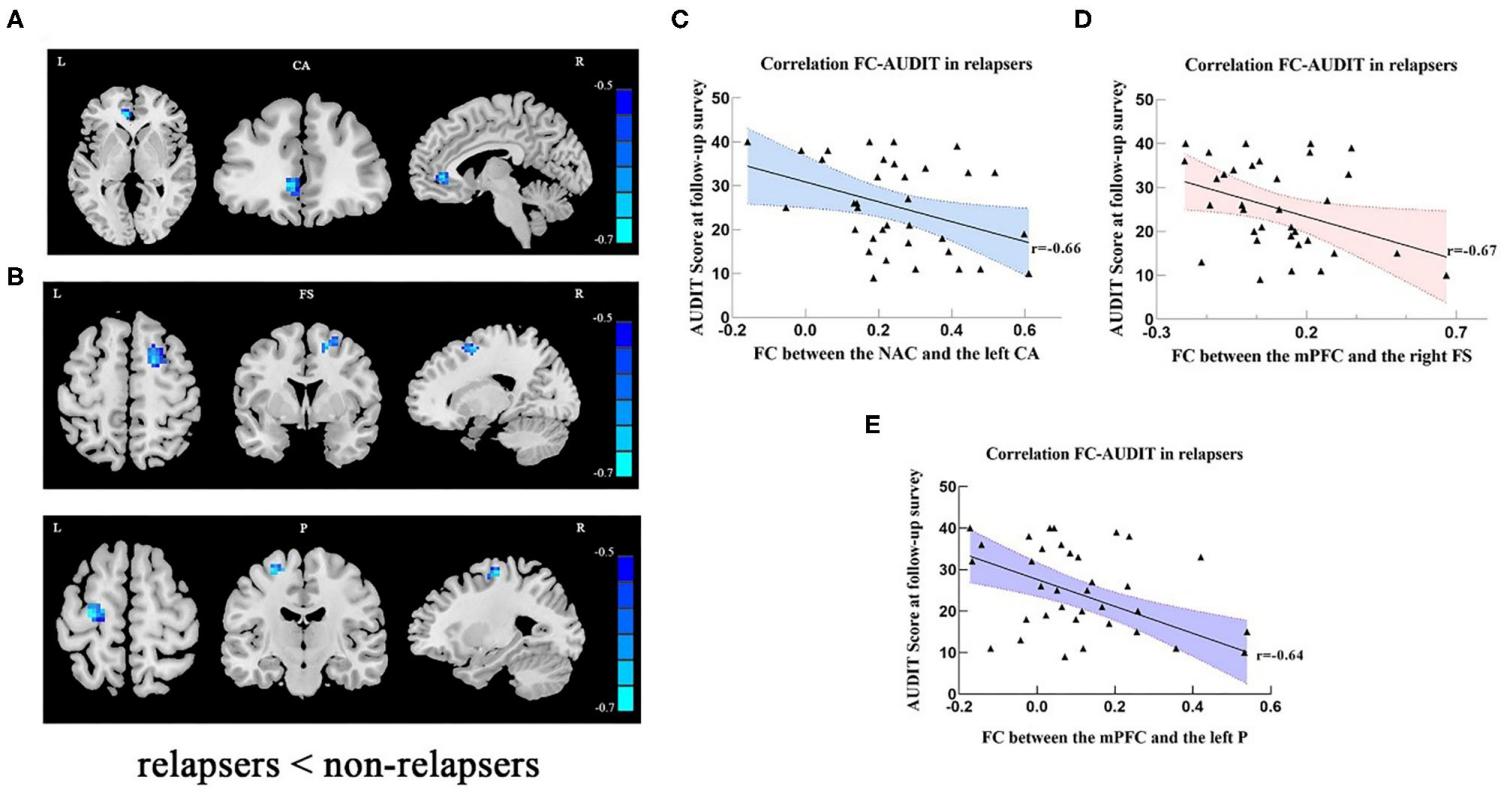
**(A)** In relapse cases, lower FC was detected in left cingulate anterior cortex (seed: NAC). **(B)** In relapse cases, lower FC was detected in right frontal superior and left precentral areas (seed: mPFC). The scatter plots show that relapse severity was negatively associated with FC between NAc and left CA **(C)**, FC between mPFC and right FS **(D)**, and FC between the mPFC and left P **(E)**. Clusters were labeled with the Automated Anatomical Labeling (AAL) atlas. The *t* maps were drawn with a threshold of *p* < 0.001 at the voxel level and *P*_FWE_ < 0.05 at the cluster level. The color bar indicates voxel-wise *t* values. lower FC is shown in blue. The brighter the blue, the lower the FC. FC, functional connectivity; NAc, nucleus accumbens; mPFC, medial prefrontal cortex; L, left; R, right; CA, cingulum anterior; FS, frontal superior gyrus; P, precentral; AUDIT, alcohol use disorder identification test.

Partial correlation analyses yielded that respective FC values of the NAc with the left cingulate anterior cortex (*r* = −0.66, x = −6, y = 39, z = 0, *P*_FWE_ < 0.001) (Figures 2A,C; Supplementary Table 5) and FC of mPFC with the right frontal superior gyrus (*r* = −0.67, x = 18, y = 3, z = 57, *P*_FWE_ < 0.001) and with the left precentral (*r* = −0.64, x = −27, y = −15, z = 63, *P*_FWE_ < 0.001) (Figures 2B,D,E; Supplementary Table 6) in the early-abstinence stage were negatively correlated with relapse severity based on the follow-up survey. Linear regression showed lower FC between the mPFC and right frontal superior gyrus, and education years were independently predictive of the severity of relapse (Table 4).

**Table 4:** Multivariate linear regression analysis to predict relapse severity (*n* = 35).

**Variables**	***R^**2**^*^a^**	**S.E**.	***t***	**β (95% CI)^b^**
Education years	0.43	0.45	−4.04	−1.84 (−2.34, −0.59)^**^
mPFC_frontal superior_R		8.07	−4.32	−34.84 (−51.44, −19.16)^***^

a *R^2^: adjusted R^2^ of determination coefficient*;

b *β (95% CI): regression coefficient (95% confidence interval) which was based on the multivariate linear regression (stepwise) using relapse severity (AUDIT score at follow-up) as the dependent variable, and age, education years, AUDIT scores at baseline survey and changes FC as independent variables; NAc, nucleus accumbens; mPFC, medial prefrontal cortex; L, left; R, right*;

** *P < 0.01*;

*** *P < 0.001*.

## Discussion

According to the hypothesis that AD is accompanied by an abnormal reward circuit and cognitive execution, which is mostly based on the findings of previous neuroimaging and neurobiological research, this study selected the NAc and mPFC as seeds to investigate resting-state FC associated with AD and its relapse after detoxification. The first part of this study is based on the baseline cross-sectional case-control data collected from a relatively larger sample (compared to previous neuroimaging studies on AD) and found that AD was associated with several lower FC values. The second part of this study is based on longitudinal data of 67 AD patients who were hospitalized and underwent detoxification and found that some lower FC values during early abstinence were predictively associated with relapse during the 6-month follow-up. These findings add to our scientific knowledge regarding neuroimaging features and biomarkers of AD and its relapse.

Regarding the lower FC between the NAc and fusiform cortex among AD patients, we propose that dysfunctional visual encoding has an influence on individuals' attention bias in the activation of the reward circuit, which could be associated with impulsive drinking behaviors in relation to cue salience. Although the defined role of the fusiform cortex is currently unclear, previous evidence suggests that its activity may be essential for encoding visual objects (such as faces, scenes, and words) and subsequent memory (41, 42). Zehra et al. found that alcohol use disorder patients showed lower activation in regions of ventral and dorsal attention networks (such as insula, parietal, and prefrontal cortex) compared with health controls (43). In addition, Hill et al. have suggested that alcohol may contribute to the hypofunctional fusiform of adolescents with a family history of AD during the execution of the eyes task of Baron-Cohen (44). Therefore, the lower FC between the NAc and fusiform cortex may indicate that the functional consistency between the reward circuit and visual encoding has been abnormally changed in AD patients. The lower FC between the mPFC and cingulum anterior may reflect the dysfunction of a crucial brain network among AD patients. A default mode network (DMN) among brain regions, including the cingula anterior, mPFC and precuneus, has been identified as the most crucial brain network in the resting state, although it is deactivated during executive task performance utilizing frontoparietal regions (45). Previous research on addiction indicated that individuals with hypoactivation in the mPFC and cingulum anterior showed deficits in response inhibition and in impulse control, which could indirectly increase the individual's susceptibility to substance use by activating stress and reward centers (46, 47). For the hypothesis of response inhibition deficits and impulse control failure in the neurobiological mechanisms underlying AD, some structural imaging studies, including a diffusion tensor imaging (DTI) study that found diminished white matter integrity in cingulate and frontal areas among individuals with AUD (48). Our evidence for lower FC between the mPFC and cingulate anterior cortex provides further functional imaging evidence. The lower FC values associated with AD found in our study are likely mostly related to the etiology of AD rather than brain impairment due to alcohol use, although a potential causal relationship between changes in brain FC and alcohol use cannot be verified because these findings are based on baseline cross-sectional case-control data.

It is especially encouraging that this study found that lower FC values between the NAc and cingulate anterior cortex and between the mPFC and calcarine sulcus, independently predicting relapse. The NAc and cingulate anterior cortex are primarily involved in processing motivational information, salience of emotion, cravings and cognitive control in the mechanism of addiction (49, 50). The calcarine sulcus plays a primary role in the visual cortex, not only accepting signals directly from neural fibers from the retina but also projecting extensively to the cerebral cortex, which is associated with recognizing text, memorizing, identifying objects, differentiating distance and determining relationship with others. Limitations in cognitive processes may cause an associated reduction in the amount of specific visual signal encoding and finally lower the probability of recognition (51, 52). Therefore, the connection of the mPFC and calcarine sulcus has an important role in recognition memory and information processing, which are involved in the reward circuit, visual cortex, and cognitive control. Previous studies have also found subtle deficits in the function of these regions that may significantly predict craving and relapse in the progression of addiction (53). We also found that relapse severity was significantly predicted by a lower education level and lower connectivity between the mPFC and right frontal superior gyrus. Lower education as a predictor of the severity of relapse is consistent with previous evidence, which indicated that those who have a lower education level have more limited cognition in relation to health issues (54). The prefrontal cortex is a core region for inhibitory control (55, 56). Its subregions, such as the mPFC, frontal superior cortex and cingulate anterior cortex, usually make differential contributions to decision making. The ventral prefrontal cortex, where the mPFC is located, mainly suppresses inappropriate risky choices, while the dorsal region including the frontal superior and cingulate anterior cortices often promotes more profitable risk-associated choices (57). Thus, it is possible that the disruption of the connection between these areas may contribute to inappropriate risky choices such as heavy drinking. Accordingly, the findings of those lower FC values during early-abstinence predictively associated with relapse and its severity are not only helpful for determining potential biomarkers for predicting AD relapse but also contribute to research on the neurobiological etiology of AD.

## Limitations

There are several limitations in the present study that were not mentioned previously. Participant's parents should be interviewed by uniform tools helpful to exclude Axis I psychiatric disorders, and we also should exclude Axis II psychiatric disorders in patients or their parents. This study was based on a priori selection of ROIs (the NAc and mPFC) as seeds, which cannot analyze FC alterations in the whole brain and may lead to observational bias. Additional whole brain network analysis studies based on larger samples should be explored to elucidate the neuroimaging perspective on mechanisms of addiction. Another disadvantage of this study is that the fMRI scan data were limited to a single scan at early-abstinence stage, which may be satisfactory for predictive association analysis but obviously not for examination of longitudinal neuroimaging changes after detoxification for both groups of relapse and non-relapse. Because relapse is considered as a return to unhealthy behaviors and negative consequences (58) this study used the optimal cutoff of the AUDIT score to screen for alcohol use disorders to define relapse. Nevertheless, some individuals characterized as non-relapsers in this study, especially those who returned to mild drinking, might relapse by the time of longer-term follow-up. Pharmacological treatment of withdrawal symptoms and complications of AD during hospitalization is complex due to varied types and doses of drugs, which may have impacts brain FC. In the current study, we did not have a sufficient sample size to control simply for those possible confounding effects. It is notable, however, that Tao et al. indicated a diversity of medicines used in AD treatment have no significant association with relapse (34). Smoking status has not been well-matched between AD cases and HCs, although smoking status was controlled as a covariate in data analysis. This is a thorny issue given the frequency co-existence of tobacco and alcohol use, and the impact of smoking on brain function is difficult to exclude entirely in this context. This study is inclusion of only male subjects, a research location socio-cultural issue compared to other international sites, due to a lack of recruitment of female participants. Becker et al. reported different changes in the brain after exposure to drugs in relation to biological sex (59). Some neuroimaging alterations associated with alcohol dependence and relapse have been found in this study. However, a further research of neurochemical changes in microscale also is mandatory.

## Conclusion

This study found that values of FC between selected seeds (i.e., the NAc and the mPFC) and some other reward- and/or impulse-control-related brain regions were associated with AD and relapse; these FC values could be potential biomarkers of AD or for predicting relapse after detoxification. These findings represent a meaningful stimulus for further research on the neurobiology of AD and other addictive disorders.

## Data Availability Statement

We are happy to share the data of the present study with other researchers for academic use on request under the regulations of the Ministry of science and technology of China, and West China Hospital of Sichuan University. Researchers may contact the primary investigators (Wan-jun Guo, guowjcn@163.com) for data sharing.

## Ethics Statement

Written informed consent was obtained from the individual(s) for the publication of any potentially identifiable images or data included in this article.

## Author Contributions

All authors listed have made a substantial, direct and intellectual contribution to the work, and approved it for publication.

## Conflict of Interest

The authors declare that the research was conducted in the absence of any commercial or financial relationships that could be construed as a potential conflict of interest.
