# Supplementary material for: Functional Connectivity of Nucleus Accumbens and Medial Prefrontal Cortex With Other Brain Regions During Early-Abstinence Is Associated With Alcohol Dependence and Relapse: A Resting-Functional Magnetic Resonance Imaging Study
Source: Front Psychiatry. 2021 Jan 28;12:609458. doi: 10.3389/fpsyt.2021.609458 (PMC7876376; doi:10.3389/fpsyt.2021.609458)
Supplement: Supplementary file 1 [file Table_1.DOCX]

| **Supplementary table 1. Changed functional connectivity between seed of NAc and other regions of whole brain among alcohol-dependence comparing with health controls.** | | | | | | |
| --- | --- | --- | --- | --- | --- | --- |
| **ROI** | **Regions** | **MNI coordinates** | | | ***t* value** | **Cluster size** |
|  |  | **X** | **Y** | **Z** |  |  |
| **NAC** | Fusiform_L | -33 | -39 | -24 | -5.27 | 154 |
|  | Temporal Superior_R | 36 | -9 | 3 | -4.53 | 47 |
|  | Temporal Superior_L | -54 | -24 | 15 | -4.76 | 147 |
|  | Postcentral_R | 9 | -24 | 48 | -5.07 | 203 |
| The *t* values were calculated with threshold at *p*<0.001 at voxel-level and *P*_FWE_<0.05 at cluster-level.  Abbreviation: NAc: nucleus accumbens; ROI: region of interest; MNI: montreal neurological institute; L: left; R: right | | | | | | |

| **Supplementary table 2. Changed functional connectivity between seed of mPFC and other regions of whole brain among alcohol-dependence comparing with health controls.** | | | | | | |
| --- | --- | --- | --- | --- | --- | --- |
| **ROI** | **Regions** | **MNI coordinates** | | | ***t* value** | **Cluster size** |
|  |  | **X** | **Y** | **Z** |  |  |
| **mPFC** | Temporal Inferior_R | 45 | -45 | -3 | -4.62 | 76 |
|  | Temporal Superior_L | -66 | -30 | 9 | -5.37 | 209 |
|  | Temporal Superior_R | 51 | -30 | 24 | -5.44 | 202 |
|  | Cingulum Middle_L | 3 | -12 | 33 | -5.46 | 219 |
| The *t* values were calculated with threshold at *p*<0.001 at voxel-level and *P*_FWE_<0.05 at cluster-level.  Abbreviation: mPFC: medial prefrontal cortex; ROI: region of interest; MNI: montreal neurological institute; L: left; R: right | | | | | | |

| **Supplementary table 3. Changed functional connectivity between seed of NAc and other regions of whole brain among relapser comparing with non-relapser.** | | | | | | |
| --- | --- | --- | --- | --- | --- | --- |
| **ROI** | **Region** | **MNI coordinates** | | | ***t* value** | **Cluster** |
|  |  | **X** | **Y** | **Z** |  |  |
| **NAC** | Cingulum Anterior_R | 9 | 39 | 6 | -3.95 | 27 |
| The *t* values were calculated with threshold at *p*<0.016 at voxel-level and *P*_FWE_<0.05 at cluster-level.  Abbreviation: NAc: nucleus accumbens; ROI: region of interest; MNI: montreal neurological institute; L: left; R: right | | | | | | |

| **Supplementary table 4. Changes functional connectivity between seed of mPFC and other regions of whole brain among relapser comparing with non-relapser.** | | | | | | |
| --- | --- | --- | --- | --- | --- | --- |
| **ROI** | **Region** | **MNI coordinates** | | | ***t* value** | **Cluster** |
|  |  | **X** | **Y** | **Z** |  |  |
| **mPFC** | Calcarine_L | -12 | -81 | 6 | -3.7936 | 154 |
| The *t* values were calculated with threshold at *p*<0.016 at voxel-level and *P*_FWE_<0.05 at cluster-level.  Abbreviation: mPFC: medial prefrontal cortex; ROI: region of interest; MNI: montreal neurological institute; L: left; R: right | | | | | | |

| **Supplementary table 5. Changed functional connectivity between seed of NAc and other regions of whole brain associated with relapse severity among relapser.** | | | | | | |
| --- | --- | --- | --- | --- | --- | --- |
| **ROI** | **Region** | **MNI coordinates** | | | ***t* value** | **Cluster** |
|  |  | **X** | **Y** | **Z** |  |  |
| **NAC** | Cingulum Anterior_L | -6 | 39 | 0 | -0.6595 | 36 |
| The *t* values were calculated with threshold at *p*<0.001 at voxel-level and *P*_FWE_<0.05 at cluster-level.  Abbreviation: NAc: nucleus accumbens; ROI: region of interest; MNI: montreal neurological institute; L: left; R: right | | | | | | |

| **Supplementary table 6. Changed functional connectivity between seed of mPFC and other regions of whole brain associated with relapse severity among relapser.** | | | | | | |
| --- | --- | --- | --- | --- | --- | --- |
| **ROI** | **Region** | **MNI coordinates** | | | ***t* value** | **Cluster** |
|  |  | **X** | **Y** | **Z** |  |  |
| **mPFC** | Frontal Superior_R | 18 | 3 | 57 | -0.6743 | 86 |
|  | Precentral_L | -27 | -15 | 63 | -0.6454 | 50 |
| The *t* values were calculated with threshold at *p*<0.001 at voxel-level and *P*_FWE_<0.05 at cluster-level.  Abbreviation: mPFC: medial prefrontal cortex; ROI: region of interest; MNI: montreal neurological institute; L: left; R: right | | | | | | |
